# Artesunate Inhibits the Development of Esophageal Cancer by Targeting HK1 to Reduce Glycolysis Levels in Areas With Zinc Deficiency

**DOI:** 10.3389/fonc.2022.871483

**Published:** 2022-05-12

**Authors:** Jing Jin, Dongli Guo, Yingying Wang, Wenpeng Jiao, Daojuan Li, Yutong He

**Affiliations:** Cancer Institute, Fourth Hospital of Hebei Medical University, Shijiazhuang, China

**Keywords:** esophageal cancer, artesunate, glycolysis, HK1, HIF-1α

## Abstract

Esophageal cancer (EC) threatens many lives in China, especially in areas with high incidences of EC. Our previous studies proved that zinc deficiency (ZD) promotes the cell cycle, thus promoting the progression of EC in areas with a high incidence of EC. Artesunate could inhibit the cell cycle, thereby inhibiting the progression of EC. In this study, we first demonstrated the mechanism by which artesunate inhibits *EC in vitro* and then demonstrated that artesunate could reverse the ZD-promoted progression of EC before EC occurred *in vivo*. The results showed that artesunate could inhibit the cell cycle, metastasis, and glycolysis of EC cells. Artesunate could target HK1, promote HK1 degradation, and reduce the levels of HIF-1α and PKM2 expression, which are key glycolysis enzymes. The *in vivo* results showed that ZD could increase the expression of HK1 and increase the incidence of EC. Artesunate reduced the incidence of EC and decreased the level of HK1 expression before EC occurred. Artesunate has an anti-EC effect by inhibiting aerobic glycolysis and has the potential to be a drug that prevents EC in areas with a high risk of EC.

## Introduction

According to Global Cancer Statistics 2020, esophageal cancer (EC) is one of the most common cancers in the world, as it has the eighth highest incidence and is the sixth leading cause of cancer-related mortality (1). Due to the lack of early symptoms, patients with EC are often already at an advanced stage when they are diagnosed. This usually leads to a poor prognosis for EC, with a 5-year overall survival rate of approximately 20% (2). The incidence of EC has significant regional differences, and areas with high incidence have more than ten-fold higher cases than the worldwide average level (3). Economic underdevelopment and nutrient deficiency are common phenomena in areas with a high incidence of EC. Zinc, as an essential element, plays a pivotal role in numerous biological processes, such as DNA repair, antioxidant defense, and cell proliferation. Zinc deficiency (ZD) is associated with a variety of disorders including retarded growth, brain development disorders, immunodeficiency, and tumorigenesis (4). Many patients with cancer, such as lungs, breast, and colorectal, have a decreased level of zinc in the blood. ZD significantly increases the risk of cancer (5–7). Zinc deficiency (ZD) has been shown to be associated with the pathogenesis of ESCC (8). Our previous studies also proved that serum ZD is related to EC (9) and that ZD promotes the cell cycle by increasing the expression of CyclinD1 and Rb, thus promoting esophageal carcinogenesis (10).

Artesunate, a water-soluble hemisuccinate derivative of artemisinin, is a remarkable antimalarial agent (11). Artesunate has been reported to have anticancer activity in many tumors, including bladder cancer (12), breast (13), prostate (14), hepatocellular carcinoma (15), and CRC (16). Our research team found that artesunate could inhibit the cell cycle, thereby inhibiting the progression of EC (17). ZD promotes the cell cycle, thus promoting esophageal carcinogenesis, and artesunate has the opposite effect as ZD. We further studied the mechanism by which artesunate inhibits the progression of EC and proved that artesunate could reverse the esophageal carcinogenesis of ZD promoted in this study.

## Materials and Methods

### Cell Culture

The human EC cell lines KYSE150 and KYSE170 were purchased from Shanghai Zhongqiaoxinzhou Biotech (China). The cells were cultured in RPMI-1640 medium (HyClone, Logan, Utah, USA) with 10% fetal bovine serum (FBS, PAN, Adenbach, Germany) at 37°C in a humidified incubator with 5% CO_2_. Artesunate was purchased from TargetMol, dissolving it in DMSO at a concentration of 20mM. The desired concentration was then matched with the medium. The final DMSO concentration was <1‰ (v/v) in all experiments.

### Cell Viability Assays

CellTiter 96^®^ Aqueous One Solution Cell Proliferation Assay kit was used to detect the viability of KYSE150 and KYSE170 cells exposed to artesunate. After inoculation in 96-well plates at a density of 1000 cells per well, the cells were incubated with the MTS tetrazolium compound for 4 h. Next, the absorbance was measured at 492 nm using a microplate reader.

### Cell Cycle Phase Distribution

Flow cytometry was used to detect the cell cycle. Briefly, after treatment with artesunate for 48 h, KYSE150 and KYSE170 cells were collected and stored in 70% alcohol for 24 h. The fixed cells were washed and resuspended with PBS, then 500 μL of propidium iodide (PI) was added to the cell suspension, incubated in the dark for 30 min, and then assessed by flow cytometry (FCM).

### Transwell Assays

The digested cells were resuspended in a serum-free medium, 1×10^5^ cells and Matrigel were added to the upper chamber, and a complete medium supplemented with serum was added to the lower chamber. After 36 h of incubation in a 37°C, 5% CO_2_ incubator, the cells were fixed with 4% paraformaldehyde and stained with 0.1% crystal violet staining solution, and the cells were counted under a microscope.

### UHPLC-Orbitrap-MS Detection

#### Sample Collection

A total of 1 mL of a mixture of precooled methanol and distilled water (four-fifths of methanol, one-fifth of distilled water) was added to the petri dish, the cells treated with artesunate were collected in a centrifuge tube with a cell scraper, the cells were centrifuged at 1000 rpm for 10 min at 4°C, the supernatant was discarded, and the cells were immediately stored in liquid nitrogen for a quenching treatment.

The quenched frozen cell samples were ground with a grinder, 300 μL of prechilled methanol was added, and the samples were vortexed and sonicated for 10 min. The samples were placed in a refrigerator at -20°C for 20 min and then centrifuged at 12000 r/min at 4°C for 10 min. The supernatant was aspirated, filtered with a microporous membrane, and placed on a machine.

#### Quality Control (QC) Sample Preparation

Ten microliters of each processed sample were mixed as a quality control sample (QC), which was used to determine the state of the instrument before injection and to balance the chromatography-mass spectrometry system, as well as to evaluate the stability of the system during the experiment.

#### Data Processing

The raw data were imported into Compound Discoverer 3.0 for data preprocessing. Blank samples were used to subtract the background and remove noise.

The preprocessed data matched the standard mass spectra of the online databases METLIN (http://metlin.scripps.edu) and HMDB (http://www.hmdb) with accurate mass and fragment information to screen metabolites. The selected metabolite data adopted the principal component analysis (PCA) model of the multivariate statistical analysis method to reflect the distribution status of the overall sample and abnormal samples, and the quality of data collection was investigated through the aggregation degree of the QC samples. The differential metabolites were determined through the VIP score of the LS-PCA model and the FC value of the volcano map, and then KEGG pathway analysis was performed on the differential metabolites.

### Seahorse Assays

After treatment with artesunate for 48 h, KYSE150 and KYSE170 cells were plated in XF96 Cell Culture Microplates at an initial cellular density of 1 × 10^4^ cells/well the day before determination. A Seahorse Extracellular Flux (XF96e) Analyzer and the Agilent Seahorse XF Glycolytic Rate Assay Kit were used to measure the extracellular acidification rate (ECAR), reflecting the glycolytic level of live EC cells. The specific experimental procedures were performed according to the manufacturer’s instructions.

### Western Blot Analysis

Cells were lysed with precooled RIPA lysis buffer on ice for 45 min, and then the lysate was centrifuged at 12000 rpm for 10 min at 4°C. The concentration of the extracted protein was determined with a BCA protein concentration determination kit, 50 μg of protein was loaded on the gel, the protein was separated by SDS–PAGE, and the protein was transferred to a PVDF membrane. The membrane was blocked with 5% skimmed milk powder at room temperature for 1 h, incubated with antibodies (anti-GAPDH, 1:10000; anti-HIF-1α, 1:500; anti-HK1, 1:500; anti-PFK-1, 1:500; anti-PKM2, 1:500) at 4°C overnight, and washed three times with 0.1% Tween-containing TBST and incubated with horseradish peroxidase-linked secondary antibody (1:2,000) for 1.5 h at room temperature. The membrane was washed three times with TBST that contained 0.1% Tween, and the immunoreactive bands were detected by a scanner.

### Molecular Docking

Glycolysis-related genes targeted by artesunate were searched through TCMSP and PharmMapper. The protein structure was downloaded from the PDB database of RCSB, PyMOL was used to remove the ligand, and AutoDock 4.2 was used to remove water molecules and add total hydrogen. The main active ingredient of artesunate was downloaded through TCMSP, it was imported into AutoDock 4.2, the total hydrogen was added and it was set as a ligand. AutoDockvina software was used to dock the ligand and the receptor and to calculate the free binding energy. The affinity and the RMSD calculated by PyMOL were used to judge the binding of the ligand and the receptor. Finally, PyMOL and Ligplot were used for visualization.

### Experiment to Induce EC in Mice

All animal experiments were approved by the Laboratory Animal Ethics Committee of Hebei Medical University Fourth Hospital. A total of 360 6-week-old C57BL/6 mice (18 ± 2 g; 1:1 male/female ratio) were obtained from Beijing Vital River Laboratory Animal Technology Co.,Ltd. (Beijing, China). The mice were randomly divided into four groups (control, ZD, artesunate, and ZD+artesunate, n=90 mice/group). The control and ZD diets were identical with the exception of zinc content, which was 60.4 and 1.6 mg/kg, respectively. Then the zinc levels in the serum of the mice with each diet were detected at the end of the experiment. All mice were treated with 4-Nitroquinoline 1-oxide (4NQO) in drinking water (100 µg/ml) from 6 weeks of age to establish an esophageal cancer model. The animals were weighed weekly and monitored daily. From each group, six mice were sacrificed at 16, 24, and 32 weeks after chemical carcinogen treatment, and at 20 and 28 weeks, 36 mice were sacrificed in each group for tumor evaluation and HK1 detection. None of the mice died unexpectedly, and all mice survived to the endpoint of the experiment.

### Immunohistochemistry

The mouse esophagus tissues were fixed in formalin and embedded in paraffin blocks. Paraffin sections (4 µm thick) were deparaffinized and rehydrated, followed by treatment with 0.02 M EDTA buffer. Sections were then immersed in 3% H2O2 and blocked with 5% normal goat serum, and then incubated with monoclonal anti-HK1 antibody overnight at 4°C. The anti-HK1 antibody was diluted in PBS buffer containing 5% normal goat serum. Negative controls for each slide were incubated with 5% normal goat serum without anti-HK1 antibody. Sections were then incubated with horseradish peroxidase-conjugated anti-rabbit IgG for 45 min at 37°C and visualized with diaminobenzidine tetrahydrochloride. Stained slides were examined under a light microscope and scored by three pathologists blinded to the clinical diagnosis.

### Statistical Analysis

The SPSS 21.0 and Excel software packages were used for the statistical analysis. Quantitative results are shown as the mean ± SD. A test-test was used for statistical analyses between the groups. P<0.05 was considered statistically significant.

## Results

### Artesunate Inhibits the Cell Cycle and Metastasis of EC Cells

**Figure 1:**
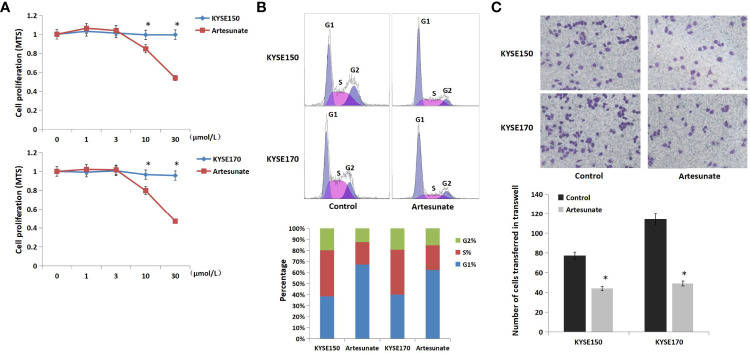
The role of artesunate in EC cells. **(A)** The effect of artesunate on the viability of EC cells by MTS. **(B)** The effect of artesunate on the cell cycle of EC cells by flow cytometry. **(C)** The effect of artesunate on the metastasis of EC cells by Transwell assays. *P < 0.05.

To evaluate the effects of artesunate on ESCC cell lines, we tested cell viability at a range of concentrations. The results showed that the cell activity was significantly inhibited in both KYSE150 and KYSE170 cells at a dose of 10 μmol/L, which might indicate cytotoxicity (**Figure 1A**). We selected 3 μmol/L as the nontoxic dose of artesunate for the subsequent experiments. We further detected the effects of artesunate on the cell cycle and metastasis of EC cells. The results showed that artesunate arrested the cell cycle at G1 and inhibited the metastasis of EC. The proportion of G1 cells increased by 75% in KYSE150 cells and increased by 56% in KYSE170 cells (**Figure 1B**). Metastasis was reduced by 43% in KYSE150 cells and reduced by 57% in KYSE170 cells (**Figure 1C**).

### Artesunate Inhibits Aerobic Glycolysis in EC Cells

**Figure 2:**
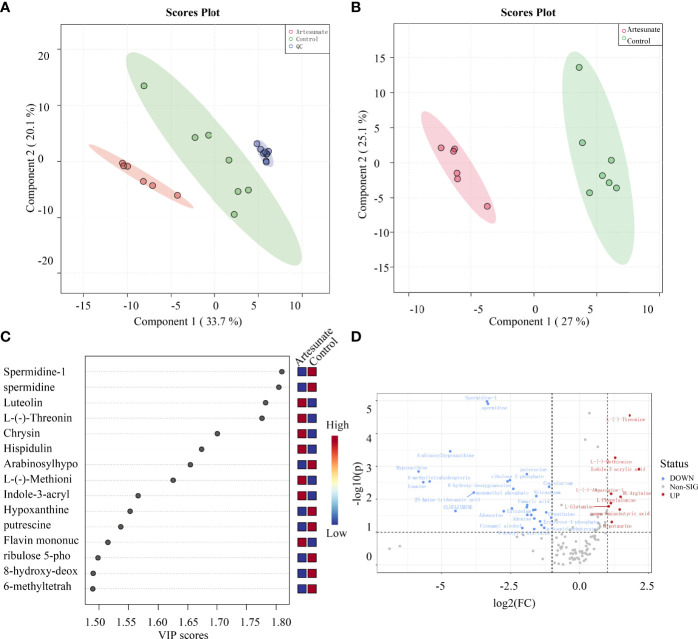
The effect of artesunate on the metabolism of EC cells. **(A)** There was a clear trend of separation between the 3 μmol/L artesunate group samples and the control group samples. **(B)** QC samples showed good aggregation. **(C)** Scoring of differential metabolites by LS-PCA. **(D)** Metabolites with significantly different levels were analyzed by volcano graphs.

The changes in metabolites with 3 μmol/L artesunate treatment were detected by UHPLC-Orbitrap-MS. There was a clear trend of separation between the cells treated with artesunate and the controls (**Figure 2A**), and all the QC samples showed good aggregation, which means there was good quality control (**Figure 2B**). We used LS-PCA to perform the scoring of differential metabolites, and combined with volcano graph analysis, we obtained 39 major differential metabolites (**Figures 2C, D**). The results showed that artesunate mainly affects 20 metabolic pathways through the analysis of these metabolites. Glycometabolism plays an important role in the progression of EC.

**Figure 3:**
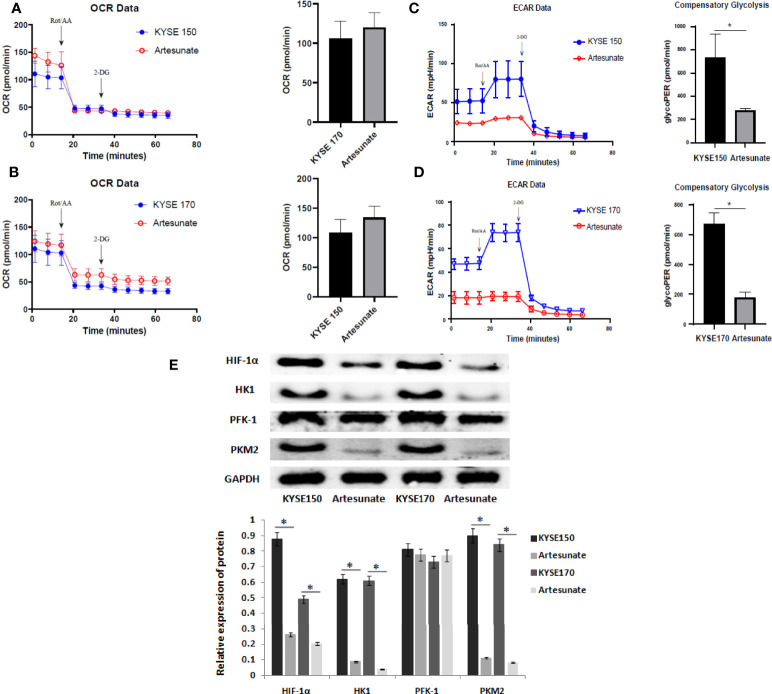
Artesunate inhibits glycolysis in EC cells. **(A)** The effect of artesunate on the mitochondrial metabolism rate was tested by OCR in KYSE150 cells. **(B)** The effect of artesunate on the mitochondrial metabolism rate was tested by OCR in KYSE170 cells. **(C)** The effect of artesunate on the glycolysis rate was tested by ECAR in KYSE150 cells. **(D)** The effect of artesunate on the glycolysis rate was tested by ECAR in KYSE170 cells. **(E)** The levels of key enzymes of glycolysis were tested by Western blotting. *P < 0.05.

Glucose metabolism includes mitochondrial metabolism and aerobic glycolysis. In this study, we tested the effects of artesunate on the level of mitochondrial metabolism in EC cells by OCR, and on the level of aerobic glycolysis by ECAR. The results showed that artesunate increases mitochondrial metabolism in EC cells, but the difference is not statistically significant (**Figures 3A, B**). Also, artesunate significantly inhibited aerobic glycolysis levels in both KYSE150 and KYSE170 cells (**Figures 3C, D**). We tested the levels of key enzymes of glycolysis by Western blotting, and the results showed that the expression levels of HIF-1α, HK1 and PKM2 were significantly reduced (**Figure 3E**).

### Artesunate Targets and Degrades HK1

**Figure 4:**
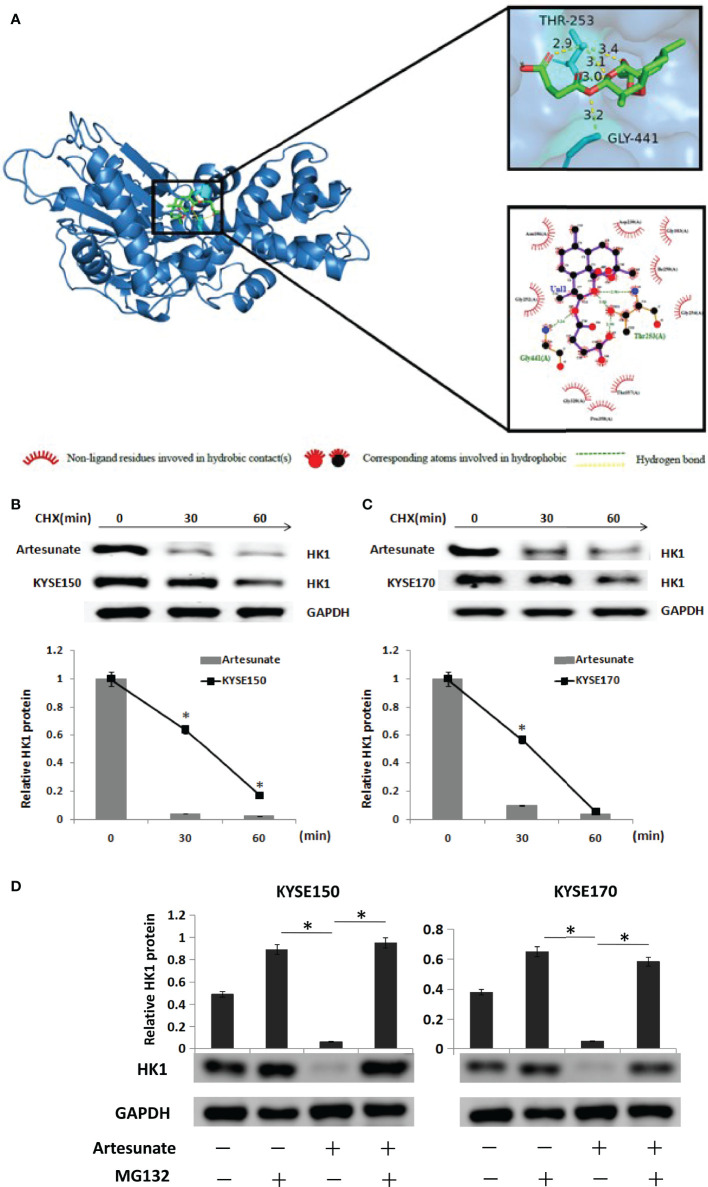
Artesunate targets and degrades HK1. **(A)** The binding site of artesunate and HK1. **(B)** The half-life of HK1protein was assessed in KYSE150 cells. Cells were treated with artesunate. **(C)** The half-life of HK1 protein was assessed in KYSE170 cells. Cells were treated with artesunate. **(D)** EC cells with artesunate treatment and control cells were treated with or without MG132 (5 μM) for 12 h. *P < 0.05.

We searched the target genes of artesunate through TCMSP and PharmMapper. In addition, we found that artesunate could target 25 glycolysis-related genes (ANG, AURKA, B3GAT3, EGFR, GALE, GLRX, ISG20, ME2, MET, MIF, PPIA, PYGL, ADH1B, ADH1C, ADH5, ALDH2, GCK, HK1, LDHB, GPI, ALDOA, PCK1, PDHB, G6PD, TPI1). HK1 is the rate-limiting enzyme of glycolysis. We simulated the interaction between artesunate and HK1 through molecular docking. Artesunate could be linked with HK1 through five hydrogen bonds located at the 253th THR and the 441th GLY (**Figure 4A**). The binding energy is -7.9 kcal/mol, which indicates that it could bind spontaneously and that the binding is stable. To determine how the HK1 protein was changed by artesunate treatment, we treated cells with cycloheximide (CHX) and analyzed the stability of HK1. The results showed that the HK1 protein degradation rate was promoted by treatment with artesunate in both KYSE150 and KYSE170 cells (**Figures 4B, C**). To elucidate the degradation pattern of HK1, cells were treated with the proteasome inhibitor MG132. The HK1 expression level did not decrease by artesunate when cells were treated with MG132, indicating that the degradation of HK1 is ubiquitin-proteasome pathway (**Figure 4D**).

### Artesunate Decreased the Expression of HK1 and Decreased the Incidence of EC in Mice

**Figure 5:**
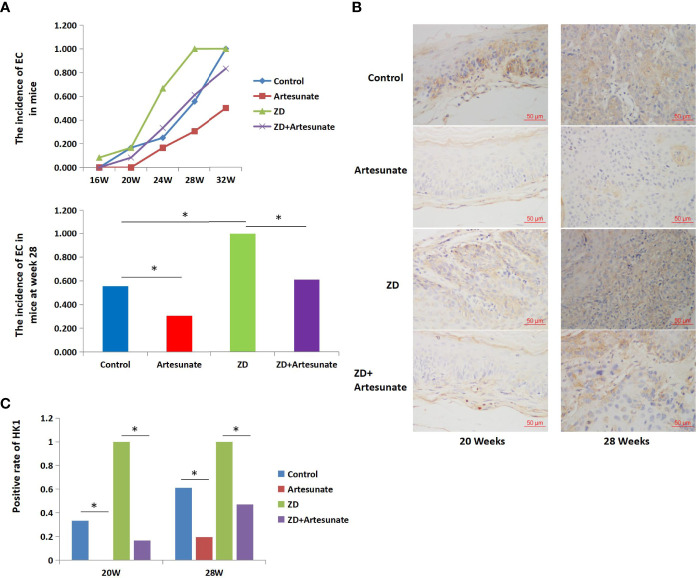
Artesunate decreased the incidence of EC and decreased the expression of HK1 in mice. **(A)** The incidence of EC in mice at several weeks after 4NQO treatment. **(B)** Immunohistochemical analysis of HK1 in the esophageal tissues of mice at weeks 20 and 28 after 4NQO treatment. **(C)** HK1-positive rate in the esophageal tissues of mice at weeks 20 and 28 after 4NQO treatment.*P < 0.05.

As shown in the experiment, all mice were administered 4NQO to induce esophageal tumorigenesis. According to the results of the previous experiment, histological examination revealed that EC was first detected at 20 weeks in mice in the control group. The cutoff date was on the 28th week after 4NQO treatment (6). The incidence of EC was significantly lower in mice treated with artesunate than in the controls at the 28th week (11/36 vs. 20/36, *P*=0.008) (**Figure 5A**). To investigate the role of artesunate, the key marker of glycolysis in the esophagus during cancer development, we performed immunohistochemistry (IHC). Both in esophageal precancerous tissues (20 weeks) and EC tissues (28 weeks), the expression levels of HK1 were lower with artesunate than in the controls (**Figures 5B, C**).

Previous studies found that ZD could promote aerobic glycolysis and the cell cycle and promote the tumorigenesis of EC. The above results showed that artesunate could inhibit glycolysis and the cell cycle. Therefore, we further studied whether artesunate can inhibit the tumorigenesis of ZD-promoted EC. We first detected serum zinc levels in mice and the results showed that ZD-diet mice had significantly lower serum zinc levels than controls (115.3 ± 33.6 μg/100ml vs. 143.1 ± 23.5 μg/100ml). The incidence of EC in mice with both ZD and artesunate (ZD+artesunate group) was significantly reduced compared with that in the ZD group and was not significantly different from that in the controls (**Figure 5A**). Both in esophageal precancerous tissues (20 weeks) and EC tissues (28 weeks), ZD increased the levels of HK1, and artesunate reduced the levels of HK1 in ZD mice and controls (**Figures 5B, C**). These results demonstrate that the levels of HK1 are decreased by artesunate before the occurrence of EC. These results indicate that the inhibition of HK1 by artesunate might be the reason for the reduced incidence of EC by artesunate.

## Discussion

Artesunate, a natural sesquiterpene extracted from a Chinese medicinal herb, was originally clinically developed for the treatment of malaria. It is considered to be suitable for drug development due to its aqueous solubility. Artesunate has also been shown to have significant antitumor activity (18). This study explores the role and mechanism of artesunate in EC. Drugs need to reach an effective concentration to function but tend to become cytotoxic at high concentrations. We first determined the nontoxic concentration of artesunate to study the mechanism against EC. Artesunate showed no cytotoxicity and exhibited an anti-EC effect in which artesunate inhibited the cell cycle and metastasis at a concentration of 3 μmol/L.

To study the mechanism by which artesunate inhibits EC, we analyzed the metabolites affected by artesunate. We found that artesunate mainly changed the levels of 39 metabolites and affected 20 metabolic pathways in EC cells. Among them, changes in glucose metabolism are closely related to cancer. There is clear evidence that mitochondria are not defective in most cancers (19). Aerobic glycolysis, the prominent feature of glucose metabolism in cancer progression, could support sustained cancer cell proliferation and malignin (20, 21). Aerobic glycolysis has also been shown to play an important role in EC (22). Therefore, we tested the effect of artesunate on aerobic glycolysis in ECs and found that artesunate could significantly inhibit glycolysis and significantly inhibit the expression of the key glycolysis proteins HIF-1α, HK1, and PKM2. We further analyzed the direct targets of artesunate in inhibiting glycolysis in EC cells and found that artesunate could bind to HK1 spontaneously and stably through hydrogen bonding and promote the degradation of HK1. HK1 is the rate-limiting enzyme of glycolysis, and artesunate inhibits glycolysis by inhibiting HK1. These results explained the mechanism by which artesunate arrested the cell cycle in the G1 phase. The G1 phase of the cell cycle is the early stage of DNA synthesis. The main function of G1 is to synthesize RNA and ribosomes, which require a large amount of energy and materials. Aerobic glycolysis can produce a large amount of ATP quickly, as well as a large number of metabolites, which can provide energy and materials for tumor progression (23). Glycolysis is inhibited by artesunate, resulting in insufficient energy and material supply, which arrested the cell cycle in the G1 phase.

Our previous studies showed that ZD could promote the pathogenesis of EC by promoting the cell cycle (10) and proved the mechanism by which ZD promotes the cell cycle (24). ZD is a common phenomenon in the high-incidence area of EC, and many studies have shown that ZD is related to the progression of EC (25, 26). ZD has also been shown to be related to aerobic glycolysis in that ZD stabilizes HIF-1α by inhibiting the ubiquitination and degradation of HIF-1α, thereby increasing aerobic glycolysis (27). In this study, our results proved that ZD could increase the expression of HK1 before the occurrence of EC. This may also be one of the mechanisms by which ZD increases the pathogenesis of EC. Correspondingly, artesunate reduced the expression of HK1 before the onset of EC and reduced the incidence of EC. Artesunate could also function this way in the case of ZD. These results proved that artesunate could inhibit the progression of EC and reverse the progression of ZD-promoted EC.

In short, it was demonstrated that artesunate has an anti-EC effect by inhibiting aerobic glycolysis. It reversed the progression of ZD-promoted EC before the occurrence of EC, indicating that artesunate has the potential to be a drug that prevents EC in areas with a high risk of EC.

## Data Availability Statement

The original contributions presented in the study are included in the article/supplementary material. Further inquiries can be directed to the corresponding author.

## Ethics Statement

The animal study was reviewed and approved by Institutional Human Ethics Committee of Hebei Medical University Fourth Hospital (Shijiazhuang, China).

## Author Contributions

YH: Study concept, Study design, Critical revision of the manuscript, responsible for all the content of the manuscript. JJ: had full access to the data in this analysis, Accrual of the participants, Drafting of the manuscript. DG, YW, WJ, and DL: Interpretation of data, Critical revision of the manuscript. All authors contributed to the article and approved the submitted version.

## Funding

This study was supported by grants from the National Natural Scientific Foundation of China (81871922) and the Hebei Province Health Department (20170745).

## Conflict of Interest

The authors declare that the research was conducted in the absence of any commercial or financial relationships that could be construed as a potential conflict of interest.

## Publisher’s Note

All claims expressed in this article are solely those of the authors and do not necessarily represent those of their affiliated organizations, or those of the publisher, the editors and the reviewers. Any product that may be evaluated in this article, or claim that may be made by its manufacturer, is not guaranteed or endorsed by the publisher.
